# Pro‐inflammatory cytokine 11 plays a pivotal role in inflammaging‐associated pathologies

**DOI:** 10.1111/acel.14360

**Published:** 2024-10-03

**Authors:** José M. Izquierdo

**Affiliations:** ^1^ Centro de Biología Molecular Severo Ochoa Consejo Superior de Investigaciones Científicas‐Universidad Autónoma de Madrid (CSIC‐UAM) Madrid Spain

**Keywords:** aging, healthspan, immunotherapy, inflammation, interleukin 11, lifespan, longevity

## Abstract

Chronic sterile inflammation contributes to aging‐associated pathologies/malignancies like cancer and autoimmune disorders. In their recent Nature article, Widjaja et al. established the pro‐inflammatory, pro‐fibrotic cytokine 11 (IL11) as a regulatory driver/hub of aging‐associated inflammation (inflammaging) in mice. Genetic and pharmacological IL11 blockade reduces inflammaging, improving healthspan, lifespan, and longevity in male and female mice, highlighting IL11 as a new inflammatory aging clock and a potential molecular target in inflammaging‐associated human degenerative diseases.

## Graphical abstract

The pro‐inflammatory and pro‐fibrotic cytokine 11 (IL11) has recently been identified as a driver/hub of regulatory dynamics and signaling pathways in aging‐associated inflammation in both male and female mice, highlighting a potential immunotherapy to advance regenerative medicine consisting of treatment with an anti‐IL11 antibody. This milestone constitutes a proof‐of‐concept of in vivo inflammatory protein blockage in mice and could reprogram some aging‐related pathologies/malignancies.

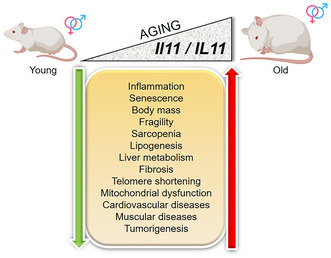

**Abbreviations**
BAT: brown adipose tissue
*Cd19*
: *Cd19* molecule
*Cd4*
: *Cd4* molecule
*Cd68*
: *Cd68* molecule
*Clstn3b*
: calsyntenin 3
CRISPR‐Cas: clustered regularly interspaced palindromic repeats‐Cas system
EGFP: enhanced green fluorescent protein
EMT: epithelial–mesenchymal transition
ERK‐p90RSK: extracellular‐signal‐regulated kinase ‐90‐kDa ribosomal S6 kinase
IgG: immunoglobulin G
IL1: interleukin‐1
*Il1*
: mouse interleukin‐1 gene
IL11: human interleukin‐1 gene/protein
*Il11ra1*
: *Il11* receptor alpha subunit
IL38: interleukin‐38
IL6: interleukin‐6
*Jak‐Stat*3: janus kinase‐signal transducer and activator of transcription signaling pathway
LKB1/STK11‐AMPK: serine/threonine kinase LKB1 (liver kinase B1)/serine/threonine kinase 11‐ AMP‐activated protein kinase signaling pathways
*Ly6C*
: lymphocyte antigen 6 family member C1
*mTOR‐p70S6K*
: mechanistic target of rapamycin kinase ‐P70 S6 kinase
*Pgc1α*
: peroxisome proliferator‐activated receptor gamma, coactivator 1 alpha
scWAT: subcutaneous white adipose tissue
*Ucp1*
: uncoupling protein 1
vWAT: visceral gonadal white adipose tissue
WAT: white adipose tissue
WT: wild type
X203: anti‐IL11 antibody

Low‐grade organ/tissue inflammation over time is associated with cellular senescence and a dysfunctional immune system, primary features of aging (López‐Otín et al., 2023). Thus, critical cellular signaling pathways are collectively altered during inflammaging, activating/potentiating major aging hallmarks (Li et al., 2023; López‐Otín et al., 2023).

Interleukins (IL1 to IL38) are produced by many different cells, with wide‐ranging effects on cell development and immune response regulation (Cook, 2023). IL11 is a 20‐kDa, extremely cationic, epithelial and stromal cell‐derived cytokine, a member of the IL6 cytokine family. IL11 was initially characterized as a hematopoietic cytokine with thrombopoietic activity, including the stimulation and proliferation of primitive stem cells. It acts in synergy with other cytokines to support the proliferation and differentiation of all hematopoietic stem cell lineages. Nevertheless, gene‐targeting with *Il11* knockout mice revealed that *Il11* is not essential for hematopoiesis in normal physiology or response to hematopoietic stress; however, it is critical within the endometrial tissue of pregnant adult female mice. Genetic deficiency of the *Il11* receptor subunit prevented decidua formation resulting in the abortion of five‐day‐old mouse embryos (Nandurkar et al., 1997; Ng et al., 2021). *Il11* receptor alpha subunit (*Il11ra*)‐null mice revealed an unprecedented role for *Il11* signaling as a “gatekeeper” of adenoma growth and possibly more advanced tumors derived from the gastrointestinal mucosa (Ng et al., 2021). The pleiotropic effect of interleukins often results in toxic effects (Cook, 2023). *Il11* administration (Oprelvekin, Neumega™) reduces the need for platelet transfusions by approximately a third in severe chemotherapy‐induced thrombocytopenia. Interleukins can improve thrombocytopenia in some chemotherapy patients but their moderate toxicity may limit their effectiveness and use as thrombopoietic agents (Cook, 2023). A transgenic *Il11*‐overexpression murine model produces animals tolerant to high hyperoxia levels (Cook, 2023). The inflammation‐promoting role of *Il11* was well established; however, *Il11* involvement in aging processes is relatively recent. Widjaja et al. demonstrated that genetic or pharmacological blockade of the *Il11* rise associated with inflammaging leads to a longer, healthier lifespan in mice (Widjaja et al., 2024).

They determined *Il11* expression in mouse liver, visceral gonadal white adipose tissue (vWAT), and skeletal muscle throughout aging; *Il11* levels were progressively upregulated in all tissues (Figure 1a). They found progressive inactivation of the LKB1/STK11‐AMPK signaling pathway and ERK‐p90RSK and mTOR‐p70S6K activation. Transgenic *Il11‐EGFP* mice were used to identify and confirm *Il11* upregulation and *Il11* expressing cells (i.e., parenchymal cells, hepatocytes, adipocytes, and myocytes) in two‐year‐old male and female mice (Figure 1a).

**FIGURE 1:**
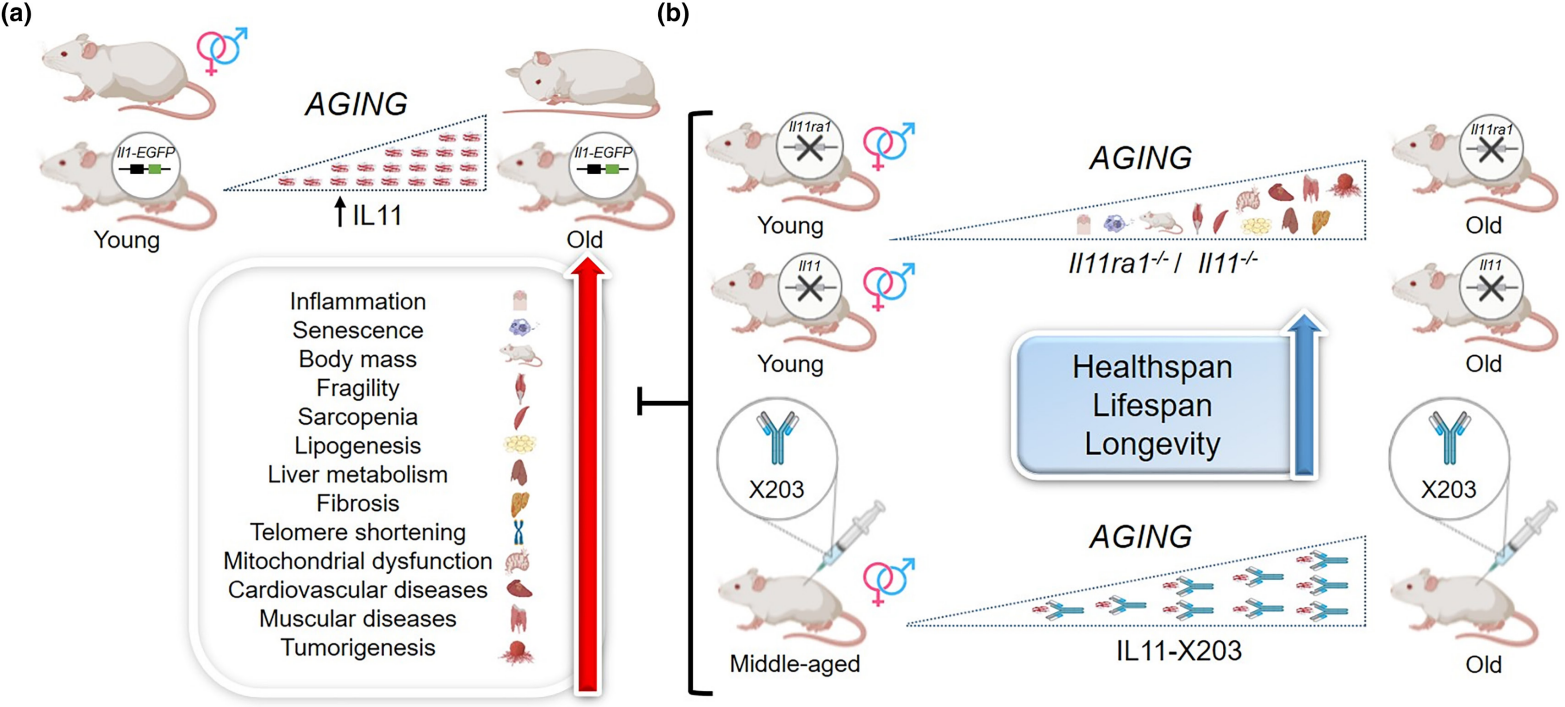
Mice live healthier and longer when inflammation‐ and senescence‐enhanced IL‐11 is blocked genetically or pharmacologically. (**a**) Schematic representation of wild type (WT) and *Ill11‐EGFP* transgenic mice to study *Il11* expression during aging. Overview of the functional impact of *Il11* upregulation on mouse pathophysiology during aging. (b) Schematic representation of *ll1ra1* and *ll11*‐null mouse models and murine supplementation of X203 (anti‐*Il11*antibody). (a, b) Samples from the aforementioned experimental mice (b) were analyzed in comparison with WT (a) to quantify various cellular and pathophysiological parameters, such as body mass, inflammatory and senescent profiling, bioenergetics, metabolism, muscular capacity, lipogenic patterns (both WAT and BAT), telomere length, mitochondrial parameters (mitochondrial DNA copy number, bioenergetics, and mitochondrial biogenesis), spontaneous tumor development, healthspan, lifespan and longevity. This figure was created with Microsoft PowerPoint and BioRender.com.

The relationship between *Il11* upregulation and senescence was explored using ten‐week‐old and two‐year‐old *Il11* receptor alpha subunit‐null mice (*Il11ra1* ^*−/−*^) and wild‐type (WT) controls (Figure 1b) (Nandurkar et al., 1997). p‐mTOR, p‐p70S6K, and p‐S6RP signaling pathway activation and canonical senescence marker levels (*p16* and *p21*) were increased in old WT mice compared with similar levels in old *Il11ra* ^*−/−*^ and young WT mice, indicating that *Il11* is associated with aging‐associated cell senescence rates (Figure 1a). They also showed that *Il11rar1* deletion reduces metabolism parameters. They verified the direct effect of *IL11* on senescence in human cell types by IL11 supplementation (Figure 1a,b). The parameters analyzed were diminished in animals lacking *Il11* compared with age‐matched controls, with values approaching those of young animals.

To verify *Il11*'s role in inflammation, senescence, and loss of metabolic function processes associated with murine aging, they generated female and male *Il11*‐deleted mouse models (Ng et al., 2021). In both young (3 months) and aged (2 years) female and male mice, they evaluated physiological traits. Results indicated that metabolic parameters improved in *Il11*‐deleted animals, with values approaching those of young animals. The most notable beneficial difference associated with loss of *Il11* function in old male mice was in WAT mass (Figure 1b).

They used a murine *Il11* neutralizing antibody (X203) to address the therapeutic benefit of *Il11* inactivation in middle‐aged male mice (Widjaja et al., 2019; Widjaja et al., 2021), analyzing whether health parameters were enhanced and if increased *Il11* expression‐associated ‘deleterious/toxic’ aging effects were reversed compared with IgG‐treated control mice. X203‐treated mice (75–100 weeks‐old) progressively lost body weight, as well as a reduction in indexed fat mass, and glucose metabolism improved; IgG had no effect. Baseline frailty scores increased slightly in all groups; however, frailty progressed in untreated/IgG‐treated but not in X203‐treated mice. Muscle strength in X203‐treated 100‐week‐old mice was greater than that in untreated/IgG‐treated age‐matched controls and of baseline values in 75‐week‐old mice. After 6 weeks of antibody administration, the respiratory exchange ratio of X203‐treated mice was higher than that of IgG‐treated mice but lower than that of young IgG‐treated mice and young untreated mice, suggesting that X203 slows age‐associated metabolic inflexibility. X203 administration was associated with higher temperatures and food intake, while locomotor activity and fecal caloric densities were similar between groups. Untreated/IgG‐treated mice had increased serum cholesterol, triglycerides, and *Il6* at the end of the study, which X203 treatment reduced to below 75‐week levels. Throughout the experiment, liver damage, liver triglycerides, and indexed liver mass markers were increased in untreated/IgG control mice, whereas they were improved or reduced in X203‐treated mice. Indexed vWAT and liver mass were reduced and indexed muscle mass increased in 100‐week‐old X203‐treated mice compared with age‐matched controls and 75‐week‐old mice. X203‐treated mice exhibited decreased subcutaneous WAT (scWAT) and increased BAT. Loss of *Il11* function reduced the age‐specific phenotype of enlarged seminal vesicles.

Fibrosis is a canonical feature of aging and a hallmark of senescence (López‐Otín et al., 2023); *Il11* is known to be profibrotic (Cook et al., 2023); therefore, fibrosis was quantified in vWAT, skeletal muscle, and liver of old mice in all experimental groups, finding a reversal of tissue fibrosis in all X203‐treated mouse organs. Thus, compared with 75‐week‐old mice, the vWAT of IgG‐treated (25 weeks) mice had increased *Il11‐*mTORC1 axis activation and senescence marker expression; contrastingly, X203‐treated mice had lower ERK‐mTOR activity and p21 and p16 expression. Untreated/IgG‐treated 100‐week‐old mice exhibited telomere shortening and reduced mitochondrial DNA copy number, which was not observed in X203‐treated mice.

They also examined the effects of anti‐*Il11* immune therapy on aging pathologies in old female mice; X203‐treated mice lost weight, while those with IgG gained weight. By the study's end, X203‐treated mice had lower fat mass, higher lean mass, and better glucose and insulin tolerance tests than at baseline, the opposite of IgG mice. Baseline frailty scores were similar between groups, progressing in IgG‐ but not X203‐treated mice. Muscle strength was greater than baseline in X203‐treated females and core body temperatures increased.

To identify the molecular mechanisms, the authors performed RNA sequencing on vWAT, gastrocnemius, and liver of 100‐week‐old IgG‐ or X203‐treated mice. In all tissues, X203‐treated mice had greater gene enrichment and clustering scores for phosphorylation and metabolism markers, whereas inflammation marker, EMT, and *Jak‐Stat3* signaling scores were decreased. Senescence‐associated gene expression in aged vWAT (The Tabula Muris Consortium, 2020), was reduced by anti‐*Il11* therapy. A similar, less‐pronounced, inhibition of liver senescence markers was observed in anti‐*Il11*‐treated mice.

The vWAT transcriptome revealed that X203 treatment highly upregulated *Ucp1*, important for the development of thermogenic ‘beige’ adipocytes in WAT depots. Upregulation of a broader ‘beiging’ program was observed in vWAT from X203‐treated mice. Proteomic data confirmed age‐dependent upregulation of *Ucp1* and *Pgc1α* in X203‐treated male mice. Additionally, an age‐related suppression of *Ucp1* expression in female control mice was attenuated in female *II11* ^*−/−*^ and *Il11ra1* ^*−/−*^ mice of both sexes. X203‐treated mice showed significant increases in mitochondrial biogenesis and function in vWAT.

*Clstn3b*, which promotes triglyceride metabolism in vWAT (Widjaja et al., 2024), was strongly upregulated in X203‐treated mice. Age‐associated *Ucp1* downregulation in BAT was limited in WT mice, however, expression was slightly increased *Il11* ^*−/−*^ mouse BAT but not in X203‐treated mice.

Proinflammatory gene expression was higher in vWAT of IgG‐treated compared with X203‐treated mice in *Il11ra1* ^*−/−*^ mouse liver and *Il11* ^*−/−*^ mouse vWAT. WT mice had higher age‐dependent proinflammatory gene expression in the vWAT than *Il11ra1* ^*−/−*^ mice of both sexes.

Stromal inflammation is associated with immune cell infiltration; in the vWAT of X203‐treated mice, immune cell surface marker genes (*Cd68*, *Cd4*, *Ly6C*, and *Cd19*) were downregulated. Histology demonstrated that the vWAT of X203‐treated mice presented a 2.5‐fold reduction in beige adipocyte foci and fewer resident CD68+ macrophages.

Longevity studies on *Il11* ^*−/−*^ and WT mice of both sexes were conducted until death; *Il11* ^*−/−*^ mice lived significantly longer than WT, especially females. *Il11* deletion extended the lifespan by an average of 24.9%.

To reach a translational perspective toward personalized medicine, they examined lifespan after *Il11* inhibition in 75‐week‐old mice receiving X203 or IgG until death; X203‐treated mice lived significantly longer (females: 25%; males: 22.5%).

Mortality in old mice is often due to carcinogenesis (Lipman et al., 2004); macroscopic autopsy revealed fewer gross tumors in *Il11*‐deleted or X203‐treated mice of both sexes.

This highlights relevant aspects that need addressing in future research: (i) are these observations on *Il11*'s role in inflammation, senescence, and inflammatory phenotype development extrapolatable to other mice lineages and mammals, including humans?; (ii) is our knowledge sufficient about *Il11* gene expression and post‐transcriptional regulation, and non‐canonical/canonical signaling pathways, to modulate its expression in a tissue/organ‐specific manner?; (iii) is *Il11* a direct (or indirect?) universal mediator through different cell signaling systems related to aging‐associated processes (irrespective of tissue/organ given its paracrine and autocrine activities)?; (iv) Phase 2 trials with IL11 neutralizing antibodies are ongoing in patients with pulmonary fibrosis: could this experience address other aging‐associated pathologies, like diabetes, cardiovascular diseases, muscle pathologies, and other autoimmunity‐based pathologies?; (v) are neutralizing antibodies against IL11 and other immune components a new senomorphic strategy, alone or combined with other drugs, to target healthy aging and avoid severe pathologies?; (vi) is serum IL11 determination a useful parameter (a new inflammatory aging clock?) to assess premature inflammaging and senescence rates and new senolytic therapies?; (vii) does this work question current cancer strategies, where guidelines recommend recombinant IL11 as a supplementary treatment to avoid chemotherapy‐induced thrombocytopenia?; (viii) similarly, should the inclusion of IL11 neutralizing antibodies be considered in current immunotherapy strategies against tumorigenesis?; (ix) previous findings have shown that IL11 is pro‐inflammatory, pro‐fibrotic, and anti‐regenerative in heart, liver, lung, and kidney diseases in mice and humans. However, other observations also demonstrated that IL11 is specifically required for appendage regeneration after trauma in some species (Cook, 2023). Given this apparent experimental paradox: how might an IL11‐neutralizing antibody strategy affect to selectively potentiate specific signaling pathways influenced by the anti‐ and pro‐regenerative functions of IL11 in the context of regenerative processes and especially the potential deleterious effects in older adults when IL11 is blocked. For instance, during wound healing or in older populations at risk for certain conditions such as myocardial infarction, where acute fibrosis is critically important?; (x) could artificial intelligence help develop synthetic molecules to block/reduce IL11 (oral statins) thus preventing deleterious processes associated with chronic pathologies through a unique multifaceted therapy?; (xi) should IL11 levels, from a certain age (55–60 years) and during treatment of age‐related pathologies, be considered for inclusion in blood tests as a preliminary step to generalize pro‐inflammatory immune patterns as personalized signatures within personalized medicine?; and (xii) could the reduction in IL11 levels be addressed by mRNA‐based therapy or CRISPR‐Cas technology, once the relevant human trials have been conducted?

To conclude, Widjaja et al. represent a good example of a study focused and conducted under the dual perspective of relevance and possible potential a priori biological sex‐dependent differences in the search for and definition of shared and/or independent therapeutic targets. Moreover, and from a purely scientific and intellectual viewpoint, this research is a pleasant surprise about the relevance of a redundant pro‐inflammatory molecule almost considered a biological and immunological vestige. Again, the magic consubstantial to scientific research emerges: what seems unimportant and is accepted as collateral, in the end, turns out to be decisive in posing new challenges with exciting prospects for achieving a healthier, longer lifespan.

## CONFLICT OF INTEREST STATEMENT

The author declares no competing interests.

## AUTHOR CONTRIBUTIONS

José M. Izquierdo: Conceptualization, visualization, writing—original draft preparation, writing—reviewing, funding acquisition and editing.

## Data Availability

N/A.

## References

[acel14360-bib-0001] Cook, S. A. (2023). Understanding interleukin 11 as a disease gene and therapeutic target. The Biochemical Journal, 480, 1987–2008. 38054591 10.1042/BCJ20220160 PMC10754292

[acel14360-bib-0002] Li, X. , Li, C. , Zhang, W. , Wang, Y. , Qian, P. , & Huang, H. (2023). Inflammation and aging: Signaling pathways and intervention therapies. Signal Transduction and Targeted Therapy, 8, 239. 37291105 10.1038/s41392-023-01502-8 PMC10248351

[acel14360-bib-0003] Lipman, R. , Galecki, A. , Burke, D. T. , & Miller, R. A. (2004). Genetic loci that influence cause of death in a heterogeneous mouse stock. The Journals of Gerontology. Series A, Biological Sciences and Medical Sciences, 59, 977–983. 15528770 10.1093/gerona/59.10.B977 PMC7110326

[acel14360-bib-0004] López‐Otín, C. , Blasco, M. A. , Partridge, L. , Serrano, M. , & Kroemer, G. (2023). Hallmarks of aging: An expanding universe. Cell, 186, 243–278. 36599349 10.1016/j.cell.2022.11.001

[acel14360-bib-0005] Nandurkar, H. H. , Robb, L. , Tarlinton, D. , Barnett, L. , Köntgen, F. , & Begley, C. G. (1997). Adult mice with targeted mutation of the interleukin‐11 receptor (IL11Ra) display normal hematopoiesis. Blood, 90, 2148–2159. 9310465

[acel14360-bib-0006] Ng, B. , Widjaja, A. A. , Viswanathan, S. , Dong, J. , Chothani, S. P. , Lim, S. , Shekeran, S. G. , Tan, J. , McGregor, N. E. , Walker, E. C. , Sims, N. A. , Schafer, S. , & Cook, S. A. (2021). Similarities and differences between IL11 and IL11RA1 knockout mice for lung fibro‐inflammation, fertility and craniosynostosis. Scientific Reports, 11, 14088. 34239012 10.1038/s41598-021-93623-9 PMC8266813

[acel14360-bib-0007] The Tabula Muris Consortium . (2020). A single‐cell transcriptomic atlas characterizes ageing tissues in the mouse. Nature, 583, 590–595. 32669714 10.1038/s41586-020-2496-1 PMC8240505

[acel14360-bib-0008] Widjaja, A. A. , Dong, J. , Adami, E. , Viswanathan, S. , Ng, B. , Pakkiri, L. S. , Chothani, S. P. , Singh, B. K. , Lim, W. W. , Zhou, J. , Shekeran, S. G. , Tan, J. , Lim, S. Y. , Goh, J. , Wang, M. , Holgate, R. , Hearn, A. , Felkin, L. E. , Yen, P. M. , … Cook, S. A. (2021). Redefining IL11 as a regeneration‐limiting hepatotoxin and therapeutic target in acetaminophen‐induced liver injury. Science Translational Medicine, 13, eaba8146. 34108253 10.1126/scitranslmed.aba8146

[acel14360-bib-0009] Widjaja, A. A. , Lim, W. W. , Viswanathan, S. , Chothani, S. , Corden, B. , Dasan, C. M. , Goh, J. W. T. G. , Lim, R. , Singh, B. K. , Tan, J. , Pua, C. J. , Lim, S. Y. , Adami, E. , Schafer, S. , George, B. L. , Sweeney, M. , Xie, C. , Tripathi, M. , Sims, N. A. , … Cook, S. A. (2024). Inhibition of IL‐11 signalling extends mammalian healthspan and lifespan. Nature, 632, 157–165. 39020175 10.1038/s41586-024-07701-9 PMC11291288

[acel14360-bib-0010] Widjaja, A. A. , Singh, B. K. , Adami, E. , Viswanathan, S. , Dong, J. , D'Agostino, G. A. , Ng, B. , Lim, W. W. , Tan, J. , Paleja, B. S. , Tripathi, M. , Lim, S. Y. , Shekeran, S. G. , Chothani, S. P. , Rabes, A. , Sombetzki, M. , Bruinstroop, E. , Min, L. P. , Sinha, R. A. , … Cook, S. A. (2019). Inhibiting interleukin 11 signaling reduces hepatocyte death and liver fibrosis, inflammation, and steatosis in mouse models of nonalcoholic steatohepatitis. Gastroenterology, 157, 777–792. 31078624 10.1053/j.gastro.2019.05.002

